# Abrasive Wear Performance of Spherical Hierarchical Structured TiC/High-Manganese Steel Composites

**DOI:** 10.3390/ma18010130

**Published:** 2024-12-31

**Authors:** Tao He, Shengnian Zhao, Dehong Lu, Yehua Jiang, Mojin Zhou

**Affiliations:** 1 Faculty of Materials Science and Engineering, Kunming University of Science and Technology, Kunming 650093, China; hetao231320@163.com (T.H.); 18298343639@163.com (S.Z.); 20190070@kust.edu.cn (M.Z.); 2 Yunnan Communications Vocational and Technical College, Kunming 650500, China; jiangyehua@kmust.edu.cn

**Keywords:** high-manganese steel, TiC particle-reinforced, spherical hierarchical structure, impact abrasive wear

## Abstract

The abrasive wear performance of TiC particle-reinforced high-manganese steel matrix composites with a spherical hierarchical structure under moderate impact energy was investigated. In the composites, TiC particles (10 μm in diameter) were concentrated within discrete spherical composite regions with diameters of about 100 μm. Impact abrasive wear tests were conducted to evaluate the wear performance of the composites with different volume fractions (30%, 40%, and 50%) of TiC particles compared with the matrix and a uniformly distributed TiC particle composite. The applied impact energy was 3 J. The results show that the hierarchical composite with 40% TiC particles exhibits the best wear resistance, with the wear rate reduced by 43.5% and 75.4% compared to the matrix steel and the uniformly distributed composite, respectively. The primary wear mechanism of the hierarchical composite is abrasive cutting. The design of the hierarchical configuration significantly enhances the material’s toughness, reducing fatigue spalling in the composite region during wear, thereby improving its wear resistance.

## 1. Introduction

Wear is a common industrial issue that often leads to shortened workpiece lifespans, resulting in significant economic losses and resource waste. Abrasive wear, in particular, is prevalent in industries such as mining, construction, and power generation, accounting for more than 50% of wear failures [1,2,3]. High-manganese steel (HMS) is a traditional wear-resistant material known for its excellent work-hardening properties and is widely used in impact wear environments. However, under low impact conditions, the work-hardening effect of HMS is limited, which subsequently diminishes its wear resistance and restricts its range of applications [4,5].

In recent years, metal matrix composites (MMCs) have garnered widespread attention due to their high specific modulus, strength, and excellent wear resistance [6,7,8,9,10]. TiC particles have become widely used as reinforcements in steel-based composites because of their excellent wettability with molten metal, high melting point, low density, and high elastic modulus [11,12,13,14]. Research shows that introducing TiC particles into a steel matrix can effectively resist wear, protect the matrix, and significantly enhance the material’s wear resistance [15]. TiC particle-reinforced steel matrix composites (TiC/steel composites) combine the excellent ductility of steel with the high strength and hardness of TiC particles, achieving hardness, strength, and wear resistance comparable to WC-Co hard alloys [16,17,18]. For instance, Deng et al. [19] used micron and nanoscale TiC particles to improve the wear resistance of low-alloy steel. Their results indicated that under both dry and wet sand conditions, the wear resistance of the TiC/steel composites was 1.5 times and 1.4 times higher than that of unmodified steel, respectively. Zhang [20] et al. prepared a TiC particle-reinforced low-alloy martensitic steel composite using the Thermomechanical Control Process (TMCP) method. Experimental results showed that under abrasive wear and impact—abrasive wear conditions, the composite exhibited superior wear resistance, with its wear resistance reaching 1.38 times and 1.60 times that of the matrix material, respectively. Similarly, Luo et al. [21] used the solidification precipitation method to prepare TiC/HMS matrix composites with varying volume fractions. Wear tests revealed that, compared to the matrix steel, the composites exhibited significantly higher wear resistance across a wide range of load conditions, reaching up to three times that of the matrix.

However, particle-reinforced metal matrix composites (PMMCs) have limitations, such as low ductility and fracture toughness [22,23,24], which hinder the improvement of wear resistance and restrict their application and adoption in the field of wear-resistant materials. For instance, Zhang et al. [25] employed a centrifugal casting process to produce WCp/Hadfield steel composite coatings, aiming to enhance the impact wear resistance of Hadfield steel under low to medium impact energy conditions. Their research demonstrates that WCp/Hadfield steel composites exhibit good wear resistance at lower impact energy levels, but the effect diminishes under higher impact energy. This is because the uniformly distributed composites possess poor toughness, making them susceptible to fracture and spalling under high-impact conditions.

To address these issues, researchers have explored methods to improve both the strength and toughness of metal matrix composites, as well as their wear resistance, by developing ceramic particle configuration distributions. Qin et al. [26] successfully fabricated a SiCp-6061Al/6061Al composite with a concrete-like structure using a vacuum pressure infiltration method. Under the same SiCp particle size and volume fraction, the resulting composite exhibited higher fracture toughness while retaining its strengthening effect. Similarly, Kou et al. [27] used gravity casting infiltration to produce Al_2_O_3_p/HMS composites with a spatial lattice structure. Compared to HMS and uniformly dispersed Al_2_O_3_p/HMS composites, the yield strength and wear resistance were both enhanced. Zhao et al. [28] fabricated a hierarchical structured composite material via pressure infiltration, where spherical TiC particle-rich hard phase regions were uniformly dispersed within TiC particle-free soft phase areas. Research demonstrates that this hierarchical structural design significantly enhances the toughness and strength of the composite, achieving 1.5 times and 8.8 times the flexural strength and impact toughness of uniformly structured composites with the same volume fraction, respectively. Although existing studies have made significant progress in optimizing the mechanical properties of composite materials, research on the wear resistance of hierarchical composites under impact–abrasive wear conditions remains limited and requires further in-depth exploration.

Based on the research of Zhao [28], this study further investigated the wear resistance of spherical hierarchical structure composites under impact abrasive wear conditions and examined the effect of the volume fraction of the composite region on their wear resistance. It explores the wear characteristics and performance of structured composites and provides a basis for the development of wear-resistant composites suitable for impact conditions.

## 2. Experimental Procedure

### 2.1. Preparation of Composite Materials

**Figure 1:**
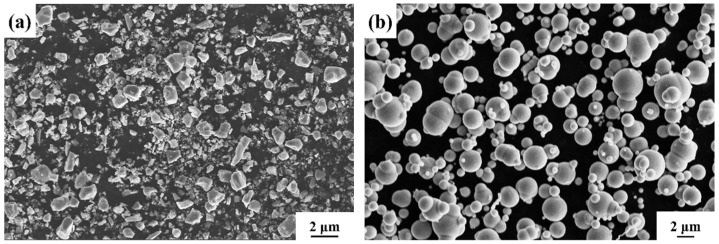

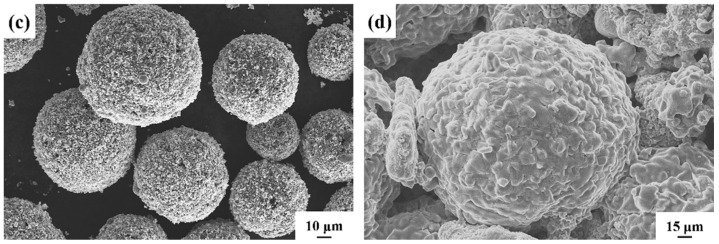
Raw materials: (**a**) original TiC particles; (**b**) reduced iron powder particles; (**c**) ceramic composite microspheres; (**d**) TiC particle preform.

The reinforcing particles selected for this study were TiC particles (Qinghe Yuanyao Alloy Products Co., Ltd., Xingtai, China, 1250 mesh) with a purity of 99.9% (the impurity is TiO_2_). Their physical properties are given in Table 1 and the average particle size is 10 μm (as shown in Figure 1a). Reduced iron powder (Qinghe Yuanyao Alloy Products Co., Ltd., Xingtai, China, 1250 mesh) with a purity of 99.9% and a particle size of 5 μm (as shown in Figure 1b) was used to adjust the TiC particle volume fraction in the preforms. Polyvinyl alcohol (PVA, Shandong Lubei Biotechnology Co., Ltd., Tai’an, China, 40%) aqueous solution with 40% mass content was used as the binder for the TiC particle preforms. HMS was selected as the matrix of the composites and its chemical composition is shown in Table 2. The selection of 14% manganese content primarily serves to form a stable austenitic microstructure, offering exceptional ductility and work-hardening capacity. The addition of 2% chromium enhances wear resistance and, in concert with manganese, contributes to grain refinement and material strengthening [29].

The preparation process for the TiC particle preform is as follows: First, TiC particles, reduced iron powder, and polyvinyl alcohol (PVA) are mixed in a mass ratio of 6:4:1. The mixture is ball milled using a planetary ball mill for 3 h with a ball-to-material ratio of 5:1, ball diameter of 10 mm, and a rotation speed of 30 rpm. Then, porous ceramic microspheres with an average particle size of 60 μm are prepared by centrifugal spray drying (shown in Figure 1c). Next, these microspheres are mixed with reduced iron powder, a 40% PVA solution, and deionized water to form a homogeneous slurry. This slurry is then poured into a pre-prepared mold (70 mm × 50 mm × 20 mm) to form the TiC particle preform. The prepared preform is first dried in a vacuum furnace for 120 min. Subsequently, the preform is transferred to a vacuum tube furnace for calcination, where the temperature is increased at a rate of 4 °C/min to 900 °C and held at 900 °C for 30 min (shown in Figure 1d).

**Figure 2:**
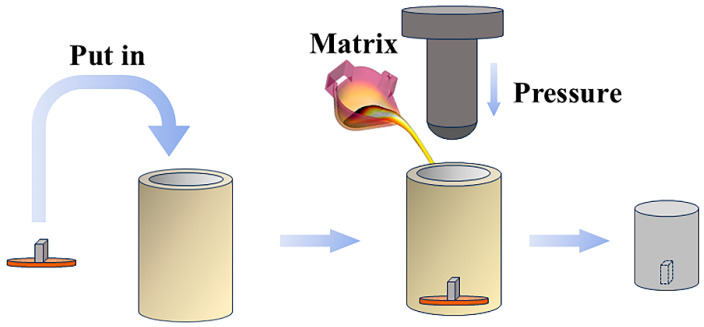
Schematic diagram of composite preparation.

Squeeze-casting infiltration technology was employed to prepare TiC/steel composites, as shown in Figure 2. The specific steps are as follows: First, the HMS was melted using an intermediate frequency induction furnace. Next, the TiC particle preform was pre-positioned in the casting mold. The molten steel was then poured into the mold at a temperature of 1550 ± 20 °C and infiltrated the preform under a pressure of 50 MPa for 3 min, which resulted in the formation of the composites. Finally, the fabricated composites underwent water toughening treatment, involving austenitization at 1050 °C for 120 min, followed by quenching in water at 20 °C.

During the preparation of the preforms, the volume fractions of the TiC particle porous microspheres were controlled at 30%, 40%, and 50% in the hierarchical composites. For comparison, the HMS matrix and uniformly distributed TiC/HMS composite with a TiC particle volume fraction of 40% were also fabricated using the same process.

### 2.2. Microstructural Characterization

Each material was machined using an electrical discharge machining (Wire EDM, GF Machining Solutions, Shanghai, China) machine to produce impact abrasive wear samples (dimensions: 10 mm × 10 mm × 30 mm) and metallographic samples (dimensions: 10 mm × 10 mm × 10 mm). Metallographic samples were prepared following standard procedures, with a 4% nitric acid–alcohol solution employed for etching the surface to reveal the microstructure. The microstructures of the powders, prefabricated blocks, and processed materials were examined using an LEO-1450 scanning electron microscope (SEM, LEO-1450, SEMTech Solutions, North Billerica, MA, USA). Furthermore, a ZEISS Crossbeam 540 (Carl Zeiss AG, Oberkochen, Germany) focused ion beam (FIB) system was employed to prepare transmission electron microscopy (TEM) samples. The composition of the micro-regions was analyzed using a JXA-iHP200F electron probe microanalyzer (EPMA; JXA-iHP200F, JEOL, Frenchs Forest, NSW, Australia).

### 2.3. Wear Performance Testing

**Figure 3:**
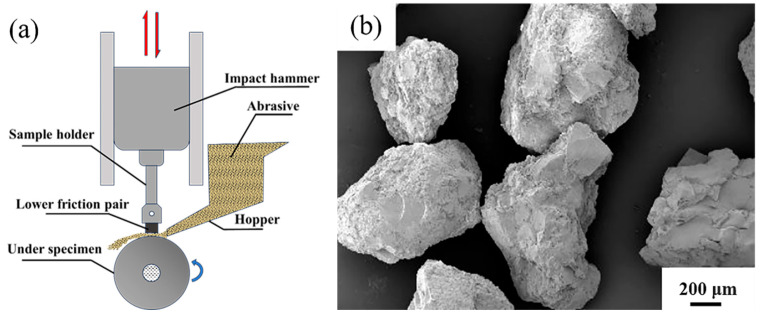
(**a**) MLD-10 impact abrasive wear machine working diagram; (**b**) abrasive morphology.

MLD-10 dynamic load wear machine was applied to carry out the impact abrasive wear tests. The experimental setup and schematic are shown in Figure 3. During the test, the sample was mounted in a fixture and along with a reciprocating impact motion, synchronized with the pendulum, was simultaneously rubbed against the rotating friction pair beneath it. The test parameters were as follows: an impact load of 3.0 J, a lower friction pair made of quenched 40Cr steel with a hardness of 55 HRC, a rotational speed of 200 r/min, and an impact frequency of 75 impacts per minute. The abrasive material is quartz sand (SiO_2_) with a particle size of 0.5–1.0 mm and a purity of 99.6% (as shown in Figure 3b), and the abrasive flow is 50 kg/h. Before the test, each sample underwent pre-grinding for 15 min, after which it was cleaned ultrasonically and weighed to determine its initial mass. Weighing was performed using an electronic balance with a precision of 1 × 10^−4^ g. The wear test consisted of six grinding durations, each lasting 20 min, for a total test period of 120 min. After each duration, the sample was weighed, and the difference in mass before and after wear was recorded as the wear loss. To ensure accuracy, samples were ultrasonically cleaned in ethanol for 10 min before each weighing.

## 3. Results and Discussion

### 3.1. Microstructure and Mechanical Properties

**Figure 4:**
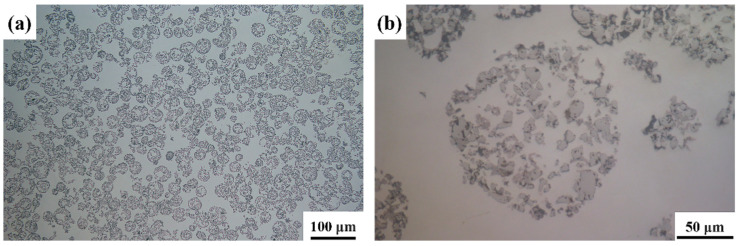

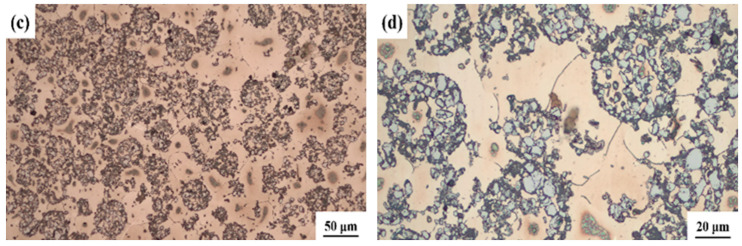
Microstructure of the hierarchical composite with 40% TiC particle volume fraction by the pressure infiltration method (**a**) at low magnification; (**b**) is the level II reinforcement; (**c**) presents the low-magnification etched metallographic image after water hardening treatment, while (**d**) shows the high-magnification etched metallographic image.

Figure 4 depicts the microstructure of a hierarchical TiC/HMS composite with a TiC particle volume fraction of 40% composite region. As shown in Figure 4a, spherical composite regions (level II reinforcements) are uniformly dispersed within the level II matrix. The bond between the spherical ceramic regions and the HMS matrix is strong, with no visible casting defects. In Figure 4b, the level I reinforcement (TiC particle) in the level II reinforcement is well-bonded to the matrix level I, and the TiC particles are relatively evenly distributed. Figure 4c,d show that the microstructure of the composite material is austenitic. Table 3 presents the bending strength and impact toughness of the experimental materials obtained from preliminary studies [26].

### 3.2. Impact Wear Performance

The mass wear data obtained from the wear tests are presented in Table 4. To accurately characterize the wear of high-manganese steel matrix, uniform composite distributed material, and hierarchical structured composite material, the mass wear loss due to wear was converted into volume wear loss. This requires knowing the densities of the various materials. In this study, the densities were determined through theoretical calculations. The method is as follows:

The density of HMS is known to be 7.900 g/cm^3^, while the density of TiC particles is 4.900 g/cm^3^. According to the Rule of Mixtures (ROM) for composites, the density of uniformly distributed composites can be calculated using the following formula:

(1) $$\rho_{uniform}V_{uniform}=\rho_{matrix}V_{matrix}+\rho_{TiC}V_{TiC}$$

For the hierarchical composites, it can be determined from empirical formulas that

(2) $$\rho_{hierarchical}V_{hierarchical}=\rho_{HMS}V_{HMS}+\rho_{uniform\;composite}V_{uniform\;composite}$$

The calculated densities for the four types of composites are as follows: 6.190 g/cm^3^ for the uniformly distributed composite with 40% TiC particle volume fraction, 7.387 g/cm^3^ for the hierarchical composite with 30% TiC particle volume fraction, 7.216 g/cm^3^ for the hierarchical composite with 40% TiC particle volume fraction material, and 7.045 g/cm^3^ for the hierarchical composite with 50% TiC particle volume fraction.

The mass of the sample before and after the impact abrasive wear test is denoted as m_1_ and m_2_, respectively. The volume wear (ΔV) of the sample is calculated using equation (3):

(3) $$\Delta V=\frac{m_{1}-m_{2}}{\rho }$$

ρ,  respectively, represents: $\rho_{uniformcomposite}$, $\rho_{hierarchicalcomposite}$, and $\rho_{HMS}$.

**Figure 5:**
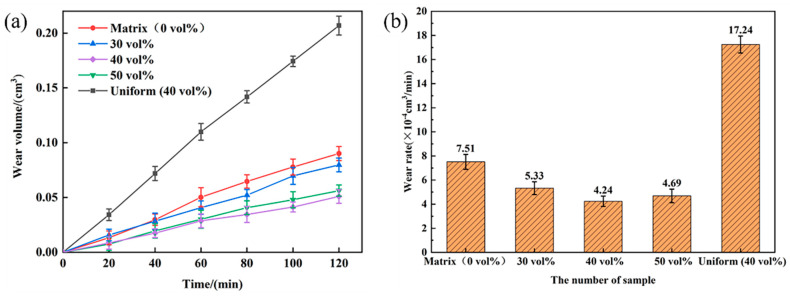
Wear resistance curves of high-manganese steel matrix, uniformly distributed composite, and hierarchical composite: (**a**) relationship curve of volume wear with time; (**b**) volume wear rate.

Figure 5 illustrates the impact abrasive wear performance of various TiC/HMS composites and HMS under 3 J impact energy. The relationship between volume wear and wear time for each sample is shown in Figure 5a. It can be observed that the volume wear generally increases continuously as the wear time extends. A linear fit of this relationship provides the wear rate, shown in Figure 5b. As evident from Figure 5b, the hierarchical composite exhibits a lower volumetric wear rate compared to both the uniformly distributed composite and the HMS matrix. Furthermore, the volumetric wear rate of the composite initially decreases and then increases with the increasing volume fraction of the composite region, forming a ‘V-shaped’ trend. The hierarchical composites show lower wear rates than the uniformly distributed composites and HMS. Notably, the hierarchical composite with a 40% TiC particle region exhibits the best wear resistance, reducing wear by 43.5% and 75.4% compared to the HMS matrix and uniformly distributed composites, respectively.

### 3.3. Impact Hardening of High-Manganese Steel Matrix

**Figure 6:**
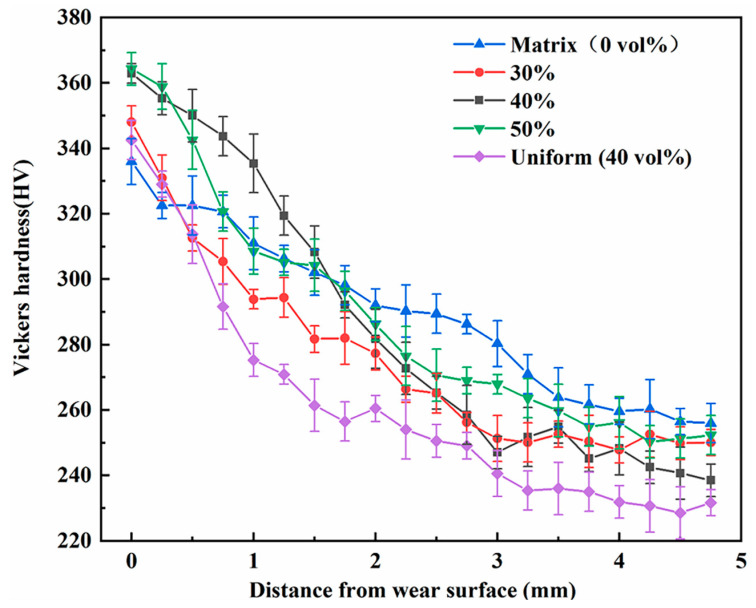
Subsurface hardness profiles of composites and matrices after impact abrasion.

The hardness of the wear surface is a critical factor in determining the wear resistance of composites. Upon impact deformation, HMS matrix undergoes work hardening, increasing surface hardness and improving wear resistance. Figure 6 illustrates subsurface hardness variations after impact abrasive wear tests. Significant work hardening is observed, as surface austenite in HMS transforms into harder martensite [32]. The hardness order is hierarchical composite with 40% TiC particle volume fraction > hierarchical composite with 50% TiC particle volume fraction > hierarchical composite with 30% TiC particle volume fraction > uniformly distributed composite with 40% TiC particle volume fraction > HMS. Hierarchical composites exhibit higher hardness and better wear resistance than uniformly distributed composites or steel matrix. As depth increases, the hardness of the composites decreases more rapidly than the matrix, which indicates that the work-hardening thickness of the composites is thinner than that of the matrix steel.

### 3.4. Wear Morphology

#### 3.4.1. Morphology of the Wear Surface of the Steel Matrix

**Figure 7:**
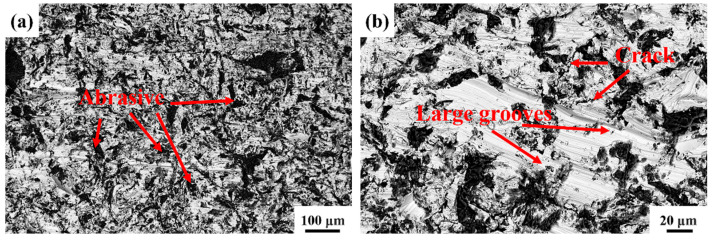
SEM micrographs of the morphology of the wear regions of the HMS matrix: (**a**) low magnification image; (**b**) higher resolution micrograph of the image shown in (**a**).

**Figure 8:**
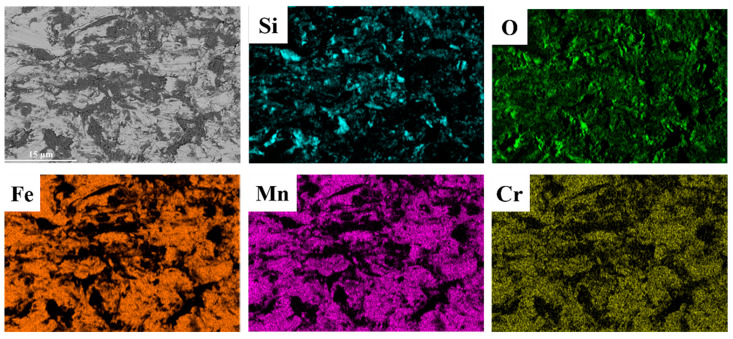
EDS analysis of HMS wear surface.

Using SEM, the wear morphology of the HMS matrix under 3 J impact energy is shown in Figure 7. Numerous grooves, caused by micro-plowing from the abrasives, are observed on the wear surface. Due to its lower hardness, the HMS matrix struggles to resist the cutting action of the abrasives, resulting in an uneven wear surface. It is also evident that abrasives are significantly embedded into the matrix under impact. Repeated impact loads cause surface hardening and brittleness, and under the combined effect of shear stress, the hardened surface layer undergoes fatigue spalling. Therefore, under 3 J impact energy, the main wear mechanisms of the HMS matrix are cutting wear and fatigue spalling [33]. Figure 8 presents the EDS analysis results of the HMS wear surface under 3 J impact energy. By comparing them with Figure 7, the black particles on the wear surface can be identified as SiO_2_ abrasives, further confirming the influence of SiO_2_ on the material surface during the wear process.

#### 3.4.2. Morphology of Wear Surface in Uniformly Distributed Composite with 40% TiC Particle Volume Fraction

**Figure 9:**
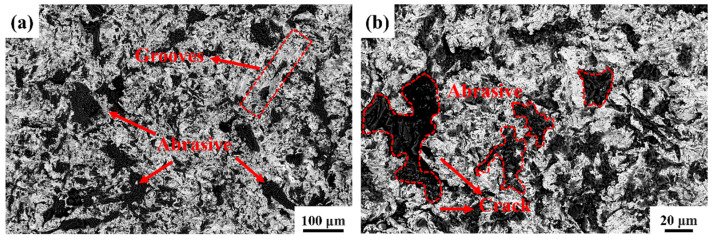
SEM micrographs of the morphology of the wear regions in a uniformly distributed composite containing 40% TiC particle volume fraction: (**a**) low-magnification, (**b**) high-magnification.

**Figure 10:**
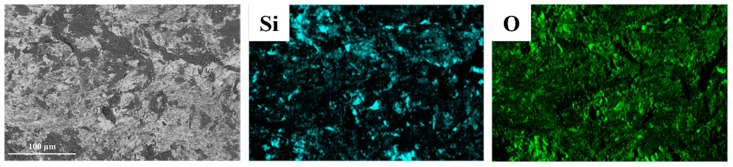

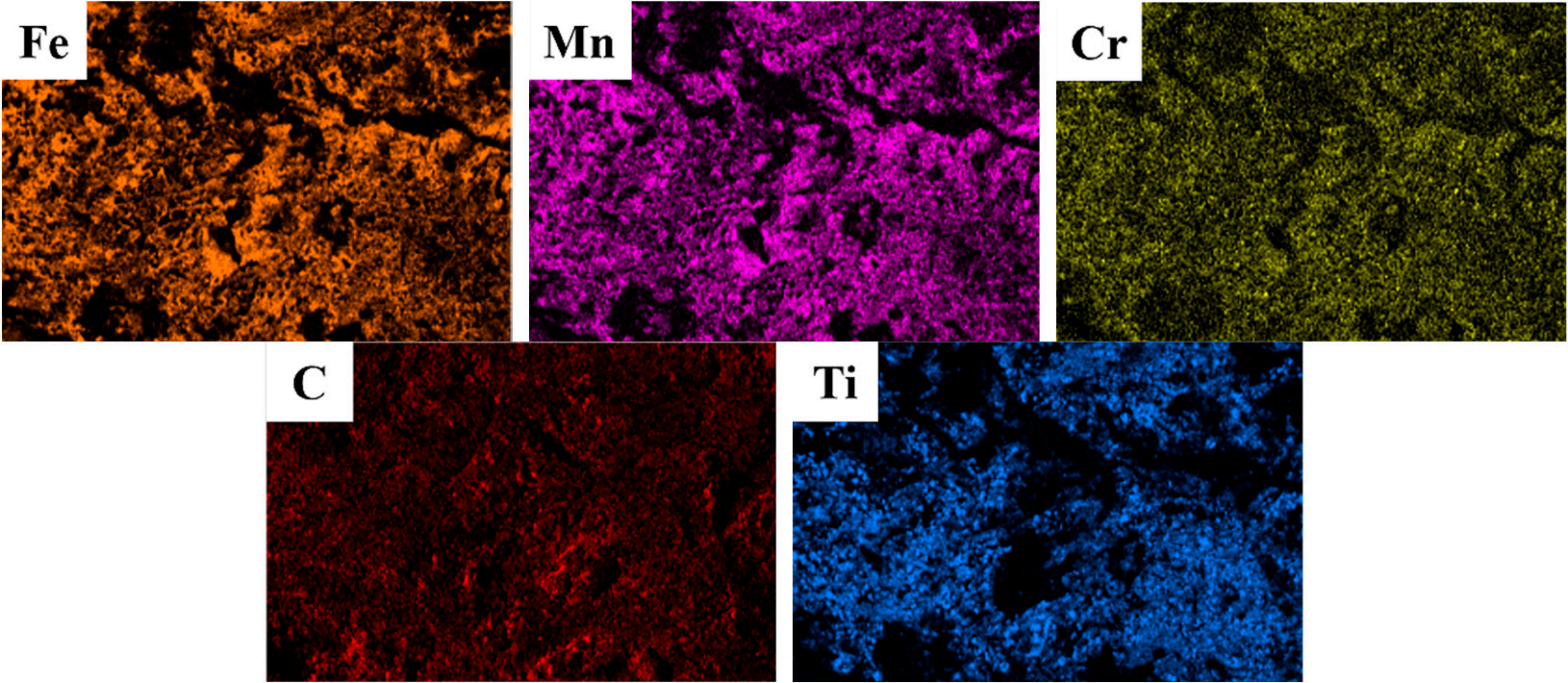
EDS analysis of wear surface of uniformly distributed composite with 40% TiC particle volume fraction.

Figure 9 presents the SEM images of the wear morphology of the uniformly distributed composite with 40% TiC particle volume fraction. Figure 10 presents the EDS analysis results of the wear surface of the uniformly distributed composite with 40% TiC particle volume fraction under 3 J impact energy. These results conclusively demonstrate that the black regions embedded in the composite are SiO_2_ abrasives. In Figure 9a, compared to the matrix in Figure 7a, the density of fine embedded abrasives on the composite surface is significantly reduced, and both the number and length of grooves are noticeably diminished. This suggests that the TiC particle reinforcement helps resist abrasive plowing, protecting the matrix. In Figure 9b, the gray areas primarily represent the HMS matrix, while the darker regions indicate the embedded abrasive particles. The embedding of these abrasive particles causes significant plastic deformation of the matrix, resulting in crack formation. Therefore, the predominant wear mechanism for the uniformly distributed composite is the embedding of abrasives, which induces material fatigue and leads to spalling.

#### 3.4.3. Morphology of the Wear Surface of Hierarchical Composites

**Figure 11:**
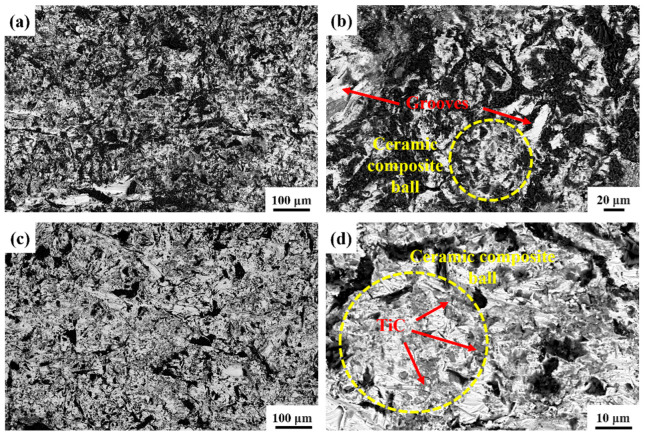

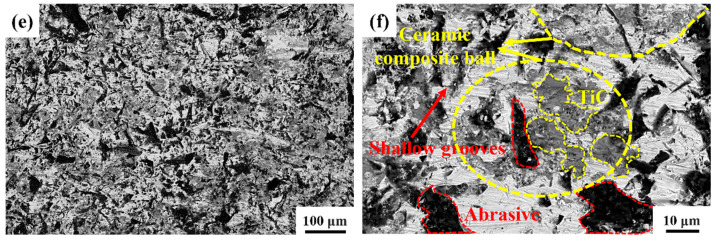
SEM micrographs showing the morphology of the wear regions in hierarchical composites with different TiC volume fractions: (**a**,**b**) for 30% volume fraction hierarchical composites, (**c**,**d**) for 40% volume fraction hierarchical composites, and (**e**,**f**) for 50% volume fraction hierarchical composites.

**Figure 12:**
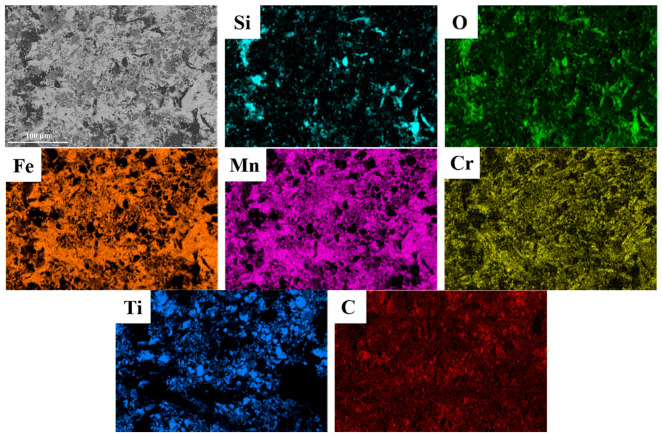
EDS analysis of wear surface of hierarchical composite with 40% TiC particle volume fraction.

Figure 11 shows SEM images of the wear surface morphology of hierarchical composite samples with TiC particle volume fractions of 30%, 40%, and 50% under a 3 J impact load. Figure 12 presents the EDS image of the wear surface of the hierarchical composite with 40% TiC particle volume fraction material after 3 J impact abrasive wear testing. All samples display visible plowing grooves and uneven pits. In Figure 11a,c,e, a significant number of abrasives are embedded in the surface, with the 30% composite showing the most and the 40% composite showing the least. Figure 11b,d,f indicate that all composites experienced plastic deformation. Around the level II reinforcement, numerous pits and embedded abrasives are observed, while the level II reinforcement itself shows fewer abrasives due to its higher hardness. The uniform distribution of TiC particles within the level II reinforcement effectively resists micro-cutting by abrasives [34].

#### 3.4.4. The 3D Morphology of the Wear Surface

**Figure 13:**
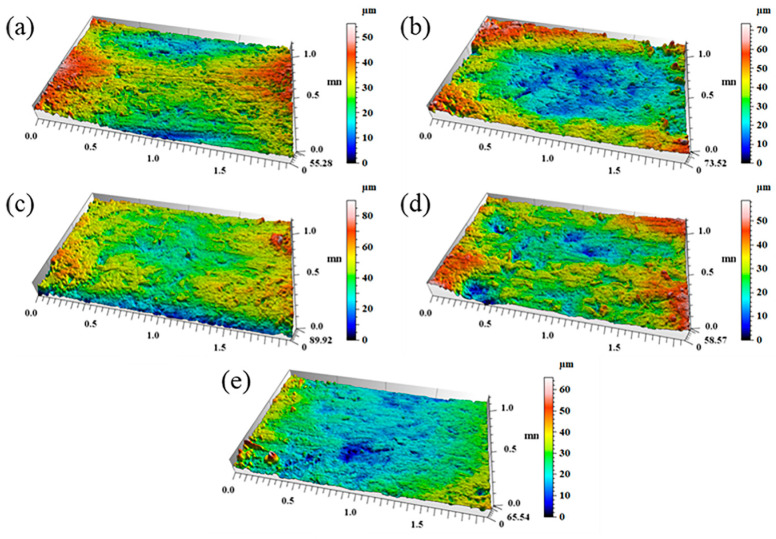
The 3D morphology of the wear surfaces of different samples: (**a**) HMS; (**b**) hierarchical composite with 30% TiC particle volume fraction; (**c**) hierarchical composite with 40% TiC particle volume fraction; (**d**) hierarchical composite with 50% TiC particle volume fraction; (**e**) uniformly distributed composite with 40% TiC particle volume fraction.

Figure 13 presents the 3D morphology maps of the wear surfaces of various samples. The 3D morphology instrument offers a more intuitive representation of the changes in surface topography during the impact abrasive wear test. By integrating data on wear rates and other metrics, the wear resistance of the composites can be further analyzed. The observations are as follows: The wear surface of the HMS matrix displays numerous plow-like plastic deformation marks, along with pronounced cutting traces, indicating poor wear resistance during the wear process (Figure 13a). The wear surface morphology of the hierarchical composite with a TiC particle volume fraction of 30% is relatively rough, suggesting a high degree of surface damage (Figure 13b). In contrast, the wear surface of the hierarchical composite with a TiC particle volume fraction of 40% is the smoothest, indicating superior wear resistance (Figure 13c). The wear surface of the hierarchical composite with a TiC particle volume fraction of 50% exhibits relatively deep pits, signifying considerable fatigue spalling during wear (Figure 13d). Finally, the wear surface of the uniformly distributed composite with a TiC particle volume fraction of 40% shows large, deep pits, suggesting extensive surface peeling and poor wear resistance (Figure 13e).

### 3.5. Subsurface Microstructure of the Wear Surface

#### 3.5.1. Subsurface Microstructure in Uniformly Distributed Composite with 40% TiC Particle Volume Fraction

To investigate the micro-deformation structure of the wear layer in this composite during impact wear, the worn samples were longitudinally sectioned perpendicular to the wear surface. After metallographic preparation, the subsurface microstructure of the samples was examined using a field emission scanning electron microscope.

**Figure 14:**
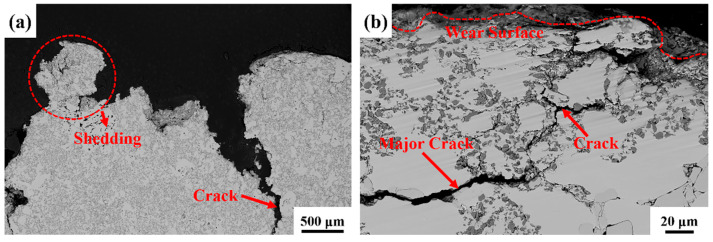

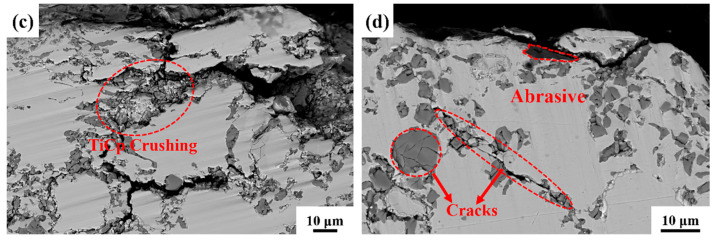
Cross-sectional view of impact wear in uniformly distributed composite with TiC particle volume fraction 40%: (**a**) illustrates the macroscopic morphology of the wear cross-section; (**b**,**c**) depict the propagation of cracks; (**d**) reveals the fragmentation of TiC particles.

In Figure 14a, it is evident that the uniformly distributed composite undergoes significant damage under a 3 J impact. Figure 14b illustrates that, upon impact, numerous cracks form within the uniformly distributed composite. These cracks propagate and bridge along the distribution path of TiC particles, ultimately leading to material failure. In Figure 14c, it can be observed that TiC particles fracture under impact, with cracks propagating outward from the fracture site, further accelerating the material’s failure process. Figure 14d demonstrates that when abrasive particles become embedded in the uniformly distributed composite, the material is directly damaged. However, the hierarchical composite exhibits a different behavior when abrasive particles are embedded.

#### 3.5.2. Subsurface Microstructure of Hierarchical Composite with 40% TiC Particle Volume Fraction

**Figure 15:**
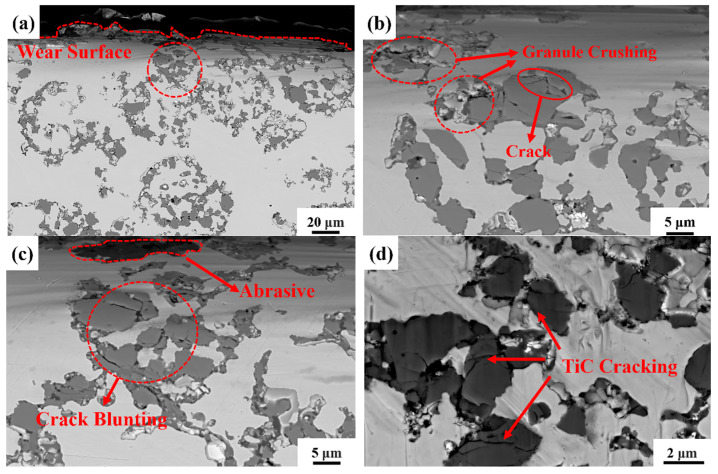
Cross-sectional view of impact wear in hierarchical composite with TiC particle volume fraction 40%: (**a**) presents the macroscopic morphology of the wear cross-section; (**b**,**c**) detail the morphology of the level II reinforcement located beneath the wear surface; (**d**) illustrates the fragmentation of TiC particles.

Figure 15 illustrates the subsurface microstructure of the composite with a TiC particle volume fraction of 40% after impact wear. In Figure 15a, it is evident that the hierarchical composite, unlike the uniformly distributed composite, is not directly damaged under the impact load. Figure 15b shows that TiC particles near the wear surface are damaged, but as the depth increases, the extent of particle damage significantly decreases. Figure 15c illustrates that when abrasives become embedded in the hierarchical composite, deformation occurs in the upper section of the level II reinforcement near the abrasive. Cracks form within the internal TiC particles, with some particles fracturing, but the cracks do not continue to propagate and instead terminate within the level II reinforcement. This behavior is attributed to the effective interaction between the level II reinforcement and the level II matrix in the hierarchical composite. Figure 15d shows the morphology of TiC particles within the level II reinforcement. Cracks in the TiC particles primarily occur in two forms: fractures through the particle and fractures along the particle boundary. When the reinforcement is subjected to severe impact, cracks tend to initiate at weak internal defect sites, leading to the fragmentation of the reinforcement phase and its subsequent detachment during the wear process.

**Figure 16:**
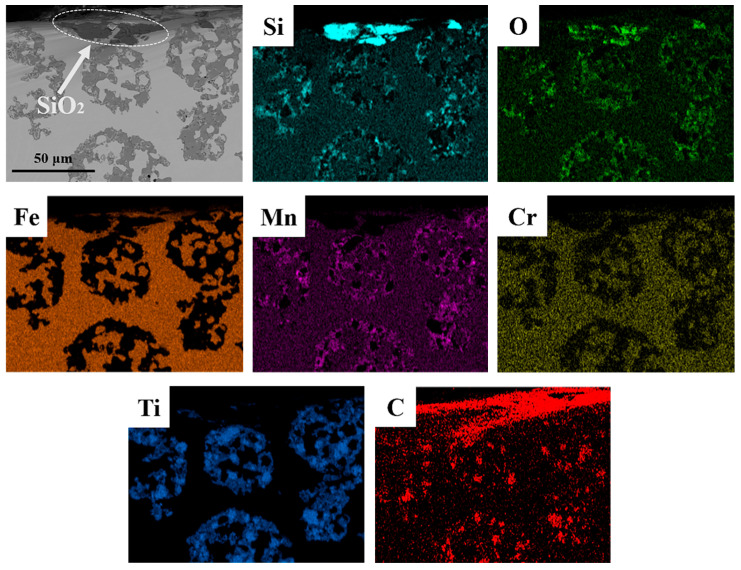
Cross-sectional EDS surface scan results of the wear surface of the hierarchical structure composite with a TiC particle volume fraction of 40%.

Combining the EDS results of the wear cross-section shown in Figure 16, it is further revealed that when fractured abrasive fragments embed into the level II matrix of the composite, stress concentration occurs in that region. If the stress exceeds the strength limit of the steel matrix, cracks may form. However, very few cracks are observed in the substrate beneath the wear surface, as the HMS substrate supporting the level II reinforcement possesses high strength, as well as good plasticity and toughness, preventing damage when abrasives penetrate.

### 3.6. TEM Testing

**Figure 17:**
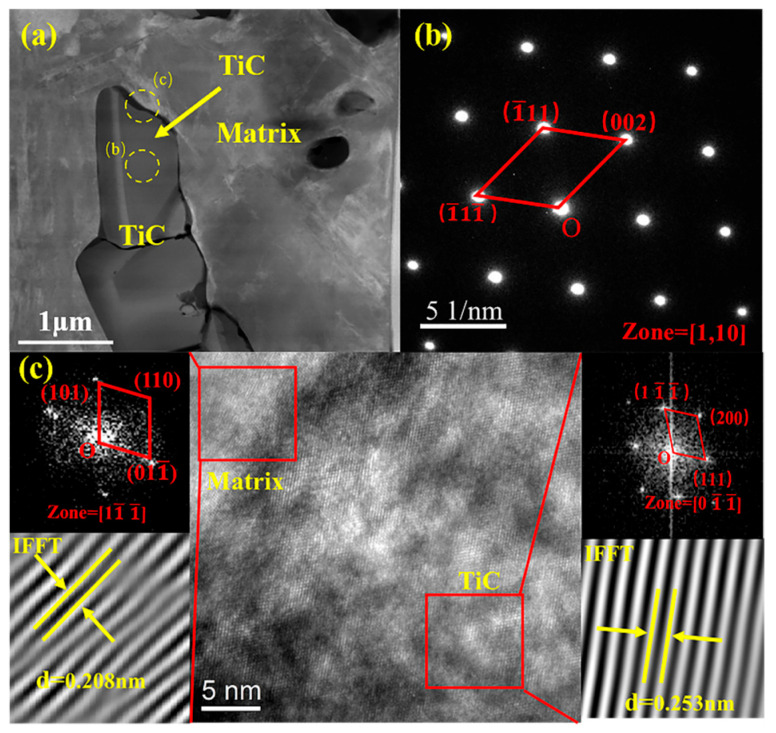
TEM (bright field) micrographs at the interfaces within the hierarchical TiC/HMS composite: (**a**) TEM bright-field image of the composite; (**b**) SADP of TiC particles; (**c**) transmission high-resolution image of the TiC–steel matrix interface.

**Figure 18:**
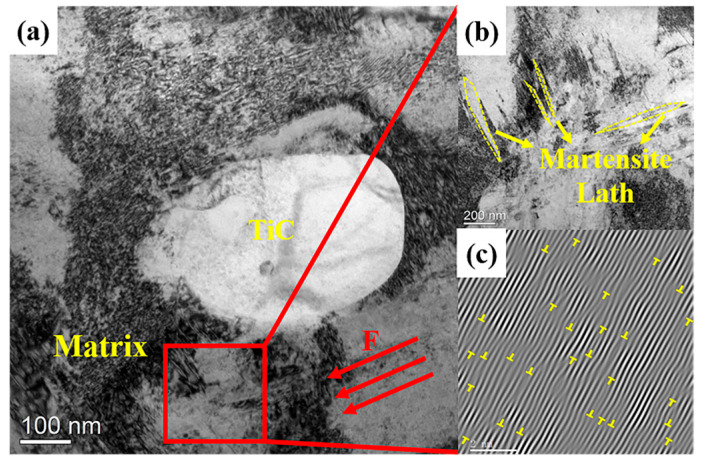
Shows the transmission electron microscope (TEM) morphology of the TiC interface in the hierarchical composite after abrasive wear testing: (**a**) TEM bright-field image of the composite; (**b**) martensitic transformation in the matrix of the red area in Figure (**a**) after impact abrasive wear; (**c**) dislocation distribution in the matrix of the red area in Figure (**a**) after impact abrasive wear.

TEM analysis was performed on the interface of the hierarchical composite with a TiC particle volume fraction of 40% after impact abrasive wear, as shown in Figure 17 and Figure 18. Figure 17a presents the morphology of the TiC/HMS interface, where no additional phases were observed. Figure 17b displays the selected area diffraction pattern (SADP) of TiC particles, confirming that TiC crystallizes in a face-centered cubic (FCC) structure, similar to that of sodium chloride (NaCl). Figure 17c shows the HR-TEM image of submicron TiC precipitates, along with their SADP. Based on the corresponding fast Fourier transform (FFT) diffraction patterns and IFFT calibration results, the interplanar spacing of the submicron TiC in Figure 17c is measured to be 0.208 nm, while the interplanar spacing of the matrix is 0.253 nm.

Figure 18a presents the microstructure near the interface between the TiC particles and the HMS matrix after the wear test. In Figure 18b, a significant amount of martensitic structure and high-density dislocations are observed around TiC particles, aligned with the direction of impact wear. This suggests that the HMS matrix underwent severe plastic deformation during the impact abrasive wear test, with some of the austenitic structures transforming into harder martensite, increasing the wear resistance of the matrix [35]. During plastic deformation, TiC particles obstruct dislocation movement, leading to dislocation accumulation at the interface (as shown in Figure 18c). The increase in dislocation density further enhances the strength of the HMS. As the HMS matrix is strengthened under impact, its load-bearing capacity for the level II reinforcement is also improved, thereby further enhancing the wear resistance of the composite [36]. Therefore, the enhanced wear resistance of TiC/HMS hierarchical composites under impact abrasive wear can be attributed to the combined effects of load transfer mechanisms and the increase in dislocation density. In summary, the wear performance of TiC/HMS composites is not solely determined by changes in the volume fraction of the reinforcement area but is closely linked to the spatial distribution of the reinforcement particles. It is the result of the interaction between the matrix region and the composite region.

### 3.7. Analysis of the Wear Resistance of Hierarchical Composites

#### 3.7.1. Comparison with Uniformly Distributed Composites

The subsurface morphology and hardness variation profile of the hierarchical composite reveal that the level II reinforcement exhibits a certain degree of toughness, allowing it to undergo some deformation under impact. The HMS matrix supporting the level II reinforcement possesses excellent plasticity, enabling it to deform quickly and absorb energy. This prevents the immediate failure of the level II reinforcement and increases the matrix’s work-hardening rate, further enhancing its load-bearing capacity for the level II reinforcement [37].

This deformation mechanism also helps control the propagation of internal cracks within the TiC particles, further protecting the ceramic particles located deeper beneath the wear surface, preventing them from collapsing and failing, as seen in uniformly distributed composites. Wear morphology analysis reveals that during the wear process, numerous pits and embedded abrasives formed by abrasive gouging appear on the surface of the matrix surrounding the level II reinforcement, while the number of pits and embedded abrasives on the level II reinforcement itself is significantly reduced. This reduction is due to the higher hardness of the level II reinforcement, making abrasive embedding more difficult. Additionally, the uniform distribution of TiC particles within the level II reinforcement effectively resists the micro-cutting action of the abrasives [34], thereby contributing to the excellent wear resistance of the hierarchical composite.

Notably, compared with uniformly distributed composites, the number of cracks generated by abrasive embedding in hierarchical composites is significantly reduced. This demonstrates that the design of the hierarchical configuration can effectively minimize the damage caused by abrasive embedding, thereby enhancing the wear resistance of the composite.

#### 3.7.2. Effect of Volume Fraction on Wear Resistance

By observing and analyzing the 3D morphology and mechanical properties of hierarchical composites with different volume fractions under 3 J impact, the impact wear performance of each sample was evaluated. For the hierarchical composite with 30% TiC particle volume fraction, the 3D morphology of the wear surface shows significant damage, which can be attributed to the lower TiC particle content leading to reduced hardness. This results in poor resistance to abrasive particles during wear, yielding inferior wear resistance. In contrast, the hierarchical composite with 40% TiC particle volume fraction exhibited the best mechanical performance among all hierarchical configurations, making it more resistant to surface damage and spalling during the wear process. As a result, the wear surface remained the smoothest, demonstrating the best wear resistance. Although the hierarchical composite with 50% TiC particle volume fraction had the highest volume fraction of level II reinforcement and hardness, its lower toughness led to deeper spalling pits on the wear surface under impact abrasive wear, reducing its overall wear resistance. Therefore, the wear resistance of hierarchical composites is not solely dependent on the volume fraction of level II reinforcement but is also closely related to the material’s mechanical properties. The hierarchical composite with 40% TiC particle volume fraction achieved the optimal balance between reinforcement content and mechanical performance, resulting in superior wear resistance.

### 3.8. Wear Mechanism

**Figure 19:**
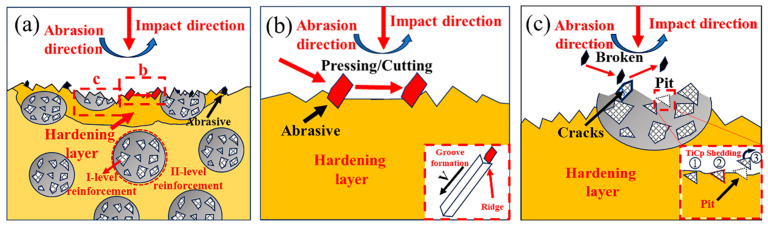
Wear mechanism diagram of the hierarchical composite: (**a**) Macroscopic wear mechanism schematic; (**b**) Wear mechanism schematic for the matrix region; (**c**) Experimental illustration of wear mechanism in the level II reinforcement zone.

Figure 19 illustrates the wear mechanism of hierarchical composites under impact abrasive wear. During the experiment, SiO_2_ abrasives flow uniformly down the upper right side of the specimen while an impact hammer applies a repetitive 3 J impact load on the composite sample. The friction pair rotates counterclockwise, bringing the abrasives into contact with the sample. Initially, the abrasives embed into the composite matrix under the impact force, and then, through the combined action of shear and tensile-compressive stresses, they cut into the composite surface. Since the HMS matrix is softer than the level II reinforcement, it undergoes cutting wear first. Once the matrix above the level II reinforcement is fully worn away, the level II reinforcement begins to play a role in resisting cutting and wear (as shown in Figure 19a). The primary wear mechanisms in the matrix region involve gouging by quartz abrasive particles (i.e., embedding and plowing) and fatigue spalling (Figure 19b). In the composite region, the wear mechanisms are characterized by abrasive chiseling and cutting, TiC particle fracture, and spalling (Figure 19c). On one hand, the high hardness of the level II reinforcement enables it to resist the abrasives while its ability to deform slightly allows it to transfer impact energy to the surrounding level II matrix. This increases the work-hardening rate of the level II matrix, improving its hardness and enabling it to better support the level II reinforcement [37]. On the other hand, the TiC particles within the level II reinforcement are encapsulated by the level I matrix, and the strong interfacial bonding makes them resistant to stripping under impact stress and abrasive forces. As wear progresses, TiC particles are exposed on the surface, where they begin to resist the micro-cutting action of the abrasives, protecting the matrix during wear and enhancing the material’s overall wear resistance.

### 3.9. Application Prospects of Hierarchical Structure Composites

#### 3.9.1. Comparison of Wear Resistance of Hierarchical Structure Composites with Other Composites

This study successfully prepared a hierarchical TiC/HMS composite material by employing fine-grained TiC particles (approximately 10 μm) in conjunction with a hierarchical design, significantly enhancing the wear resistance and toughness of the TiC/HMS composite material in impact abrasion environments. When the volume fraction of TiC particles reached 40%, the composite exhibited optimal wear resistance and mechanical properties, with its wear resistance improving by approximately 1.8 times compared to the HMS matrix. Additionally, compared to uniformly distributed TiC particle composite materials with the same volume fraction, the impact toughness of the hierarchical composite increased by 8.8 times, and the bending strength improved by 1.5 times. In contrast, the uniform distribution WC-reinforced high-manganese steel composite prepared by Zhang [25] et al. using centrifugal casting showed a significant decline in wear resistance with increasing impact energy, performing well at low impact energies but exhibiting a significant reduction in wear resistance as impact energy increased. Wang [38] et al. prepared a ZTA-reinforced high-manganese steel composite using the gravity infiltration method, which demonstrated the best wear resistance at 1 J impact energy but only 1.1 times that of the matrix at 3 J impact energy. On the other hand, Zhang [20] et al. prepared a TiC particle-reinforced low-alloy martensitic steel composite using thermomechanical controlled processing, achieving an optimal wear resistance of 1.6 times that of the matrix material at 3 J impact energy. These comparative studies demonstrate that the hierarchy design in this research effectively overcomes the issue of reduced wear resistance in uniformly distributed composites under high impact conditions and offers significantly better wear resistance enhancement compared to the matrix, providing important theoretical support and application prospects for developing composites with both high toughness and high wear resistance.

#### 3.9.2. Application Scenarios of Hierarchical Composite Materials

This study is aimed at enhancing the performance of HMS in high-impact load conditions commonly encountered in industries such as mining, construction material production, and metallurgy. By introducing TiC particles and incorporating a hierarchical composite design, this research aims to further improve the material’s strength, toughness, and overall wear resistance.

Taking the jaw plate of a jaw crusher as an example, this component requires high resistance to repeated impact and abrasion during operation. Therefore, it demands materials with superior impact resistance, hardness, and wear resistance. Although HMS is widely used in jaw plate materials due to its excellent work-hardening properties, it frequently exhibits insufficient work hardening during the initial application phase, leading to significant wear and severe deformation. This phenomenon significantly shortens its service life and complicates subsequent replacement and maintenance, thereby limiting its application scope in certain operating conditions.

To address these issues, the TiC/HMS hierarchical composite material developed in this study not only improves wear resistance but also maintains the high strength and good toughness of HMS, thereby offering better application prospects. Furthermore, the composite material developed in this study has excellent potential for use in other similar high-impact abrasion conditions. For instance, components such as the hammerhead of a hammer crusher, the bowl liner of a cone crusher, and the liner of a large autogenous mill all require materials with high hardness, high toughness, and superior impact and wear resistance. By adopting this composite material, the service life and performance of these components can be significantly enhanced, leading to reduced operational costs and increased productivity.

## 4. Conclusions

In this study, TiC/HMS hierarchical composites were successfully fabricated using pressure infiltration. The microstructure, mechanical properties, and impact–abrasive wear resistance of the composite were investigated and compared with uniformly distributed composites and the HMS matrix. The following conclusions were drawn:

(1) In the 3 J impact–abrasive wear tests, a comparison of TiC/HMS hierarchical composites with different composite region volume fractions reveals that the composite with a 40 vol.% composite region exhibited superior wear resistance. This performance aligns with the tested mechanical properties of the material.
(2) A comparison of the wear rates between hierarchical composites and uniformly distributed composites revealed that, for the same volume fraction, the hierarchical composites exhibit significantly better wear resistance. Specifically, the wear rate of the hierarchical composite with 40% TiC particle volume fraction was 43.5% lower than that of the HMS matrix and 75.4% lower than that of the uniformly distributed composites.
(3) An analysis of the subsurface crack propagation and mechanical properties of the TiC/HMS hierarchical composite reveals that, compared to uniformly distributed composites, under impact, the II-level reinforcement undergoes some deformation when abrasive particles intrude due to its toughness. Meanwhile, the HMS matrix supporting the II-level reinforcement exhibits excellent plasticity, enabling it to deform and absorb energy in a timely manner. This prevents the immediate failure of the II-level reinforcement, halts the outward propagation of internal cracks within the TiC particles, and protects the deeper ceramic particles beneath the wear surface.

## Figures and Tables

**Table 1:** Physical properties of TiC [30].

Type of Carbide	Crystal Structure	Microhardness (GPa)	Melting Point (°C)	Coefficient of Thermal Expansion (×10^−6^/°C)	Thermal Conductivity (W/m·K)	Density (g/cm^3^)	Wettability Angle with Molten Iron (°)
TiC	FCC	28–35	3067	7.4	21.0	4.91	28

**Table 2:** Chemical composition of HMS (wt.%) [31].

C	Mn	Cr	Si	S	P	Fe
1.4	10–14	2.0	0.4	<0.05	<0.1	Bal.

**Table 3:** Mechanical properties of high-manganese steel matrix, uniform composite distributed material, and hierarchical structured composite material [28].

Volume Fraction (%)	Hardness (HRC)	Bending Strength (MPa)	Impact Toughness (J/cm²)
0	19.3	1296.10	284.0
30	52.9	711.60	15.0
40	57.0	907.97	21.2
50	60.7	765.53	19.9
40 uniform	52.9	609.8	3.4

**Table 4:** Mass loss in the abrasive wear process under impact was investigated for the high-manganese steel matrix, uniform composite distributed material, and hierarchically structured composite material.

Time (min)	Uniform 40% (g)	Matrix (g)	Hierarchical 30% (g)	Hierarchical 50% (g)	Hierarchical 40% (g)
20	0.212	0.104	0.116	0.052	0.061
40	0.444	0.234	0.209	0.137	0.125
60	0.680	0.396	0.300	0.212	0.207
80	0.877	0.510	0.384	0.286	0.248
100	1.078	0.614	0.514	0.337	0.298
120	1.280	0.711	0.588	0.396	0.367

## Data Availability

The original contributions presented in this study are included in the article. Further inquiries can be directed to the corresponding author.
